# High quality of SARS-CoV-2 molecular diagnostics in a diverse laboratory landscape through supported benchmark testing and External Quality Assessment

**DOI:** 10.1038/s41598-023-50912-9

**Published:** 2024-01-16

**Authors:** John Sluimer, Willem M. R. van den Akker, Gabriel Goderski, Arno Swart, Bas van der Veer, Jeroen Cremer, Ngoc Hoa Chung, Richard Molenkamp, Jolanda Voermans, Judith Guldemeester, Working Group SARS-CoV-2 Diagnostics The Netherlands, Annemiek van der Eijk, Menno D. de Jong, Glen Mithoe, Mirjam H. A. Hermans, Jessica L. de Beer, Els Wessels, Christian von Wintersdorff, Suzan Pas, Jaco J. Verweij, Willem J. G. Melchers, Jeroen H. B. van de Bovenkamp, Ali Vahidnia, Lilli Gard, Rob Schuurman, Bas Wintermans, Maurine Leversteijn-van Hall, Paul Smits, Theun de Groot, Birgit A. L. M. Deiman, Aldert Bart, Wil van der Reijden, Sanela Svraka-Latifovic, Adri G. M. van der Zanden, Steven Thijsen, Rainer Schubbert, Lisa L. Dreesens, Gert van Duijn, David S. Y. Ong, Monique Oostra, Sylvia Bruisten, Marijke van Trijp, Annika Pettersson, Nathalie D. van Burgel, Joke Oudbier, Michael van der Linden, Michiel van Rijn, Martine P. Bos, John Rossen, Theo A. Schuurs, Roger Grosser, Pieter Smit, Roel H. T. Nijhuis, Wouter Rozemeijer, Thijs van de Laar, Theodoor M. M. Scheepers, Leonard C. Smeets, Jacky Flipse, Bertie de Leeuw, Remco Dijkman, Noortje van Maarseveen, Marco Koppelman, Bent Postma, Erik J. van Hannen, Khoa Thai, Kathrin Braun, Raf J. F. Schepers, Jana Henning, Eva R. van Hees, Mirriam G. J. Tacken, Jaqueline Mol, Edou R. Heddema, Dirk Eggink, Lance D. Presser, Adam Meijer

**Affiliations:** 1 Centre for Infectious Disease Control, National Institute for Public Health and the Environment (RIVM), Bilthoven, The Netherlands; 2 Department of Viroscience, Erasmus University Medical Center, Rotterdam, The Netherlands; 3 Working Group SARS-CoV-2 Diagnostics The Netherlands, Bilthoven, The Netherlands; 4 Department of Viroscience, Clinical Virology, Erasmus University Medical Center, Rotterdam, The Netherlands; 5 Department of Medical Microbiology and Infection Prevention, Amsterdam University Medical Center, Amsterdam, The Netherlands; 6 Medical Microbiology, Certe Laboratory for Infectious Diseases, Groningen, The Netherlands; 7 Department of Medical Microbiology and Infection Control, Jeroen Bosch Hospital, ’s-Hertogenbosch, The Netherlands; 8 Laboratory of Microbiology Twente Achterhoek (LabMicTA), Hengelo, The Netherlands; 9 Department of Medical Microbiology, Leiden University Medical Center, Leiden, The Netherlands; 10 Department of Medical Microbiology, Infectious Diseases and Infection Prevention, Maastricht University Medical Center, Maastricht, The Netherlands; 11 Microvida Location Bravis, Roosendaal, The Netherlands; 12 Laboratory for Medical Microbiology and Immunology, Microvida Elisabeth-Tweesteden Hospital, Tilburg, The Netherlands; 13 Department of Medical Microbiology, Radboud University Medical Center, Nijmegen, The Netherlands; 14 Laboratory of Medical Microbiology, PAMM, Veldhoven, The Netherlands; 15 Regional Public Health Laboratory Kennemerland, Haarlem, The Netherlands; 16 Medische Microbiologie and Infectiepreventie, Universitair Medisch Centrum Groningen, Groningen, The Netherlands; 17 Department of Medical Microbiology, University Medical Centre Utrecht, Utrecht, The Netherlands; 18 Department of Medical Microbiology and Infection Control, ADRZ Admiraal de Ruyter Ziekenhuis, Goes, The Netherlands; 19 Department of Medical Microbiology, Alrijne Ziekenhuis, Leiden, The Netherlands; 20 Eurofins Hoog Volume Laboratorium, Rijswijk, The Netherlands; 21 Laboratory of Medical Microbiology Medische Microbiologie, Section Molecular Biology, Atalmedial, Amsterdam, The Netherlands; 22 Department Medical Microbiology and Infectious Diseases, Canisius Wilhelmina Ziekenhuis, Nijmegen, The Netherlands; 23 Clinical Laboratory, Catharina Ziekenhuis Eindhoven, Eindhoven, The Netherlands; 24 Department of Medical Microbiology, Tergooi MC, CBSL, Hilversum, The Netherlands; 25 Laboratory for Medical Microbiology, Comicro BV, Hoorn, The Netherlands; 26 Laboratory for Medical Microbiology and Infection Prevention, Deventer Ziekenhuis, Deventer, The Netherlands; 27 Diagnostiek Voor U, Eindhoven, The Netherlands; 28 Department of Medical Microbiology, Laurentius Ziekenhuis, Roermond, The Netherlands; 29 Department of Medical Microbiology, Viecuri Medisch Centrum, Venlo, The Netherlands; 30 Department Medical Microbiology and Immunology, Diakonessenhuis Utrecht, Utrecht, The Netherlands; 31 Eurofins Genomics Europe Applied Genomics GmbH, Ebersberg, Germany; 32 Clinical Laboratory, Eurofins Nederlands Moleculair Diagnostisch Laboratorium B.V, Rijswijk, The Netherlands; 33 Fenelab Consortium, MasterLab B.V. - Nutreco Nederland B.V., Boxmeer, The Netherlands; 34 Fenelab Consortium, Mérieux NutriSciences, Ede, The Netherlands; 35 Fenelab Consortium, NofaLab B.V., Schiedam, The Netherlands; 36 Fenelab Consortium, Normec Biobeheer, Rotterdam, The Netherlands; 37 Fenelab Consortium, NutriControl B.V., Veghel, The Netherlands; 38 Fenelab Consortium, Nutrilab B.V., Giessen, The Netherlands; 39 Fenelab Consortium, SGS Nederland B.V., Spijkenisse, The Netherlands; 40 Fenelab Consortium, Triskelion, Utrecht, The Netherlands; 41 Department of Medical Microbiology and Infection Control, Franciscus Gasthuis and Vlietland, Rotterdam, The Netherlands; 42 Department of Medical Microbiology, Gelre Ziekenhuizen Apeldoorn, Apeldoorn, The Netherlands; 43 Department of Infectious Diseases, GGD Amsterdam, Public health laboratory, Amsterdam, The Netherlands; 44 Department of Medical Microbiology and Infection Prevention, Groene Hart Ziekenhuis, Gouda, The Netherlands; 45 Department of Medical Microbiology, Haaglanden Medisch Centrum locatie Westeinde, Den Haag, The Netherlands; 46 Department of Medical Microbiology, Hagaziekenhuis, Den Haag, The Netherlands; 47 How Are You Diagnostics, Lelystad, The Netherlands; 48 Department of Medical Microbiology, IJsselland Ziekenhuis, Capelle aan den IJssel, The Netherlands; 49 Department of Medical Microbiology, Ikazia Ziekenhuis, Rotterdam, The Netherlands; 50 inBiome, Amsterdam, The Netherlands; 51 Laboratory of Medical Microbiology and Infectious Diseases, Isala Ziekenhuis, Zwolle, The Netherlands; 52 Izore – Centrum Infectieziekten Friesland, Leeuwarden, The Netherlands; 53 Labor Dr. Wisplinghoff, Köln, Germany; 54 Maasstad laboratorium, Molecular Diagnostics Unit, LMM, Maasstad Ziekenhuis, Rotterdam, The Netherlands; 55 Laboratory for Medical Microbiology and Medical Immunology, Meander Medisch Centrum, Amersfoort, The Netherlands; 56 Laboratory for Medical Microbiology, Noordwest Ziekenhuisgroep, Alkmaar, The Netherlands; 57 Laboratory for Medical Microbiology, OLVG Lab B.V., Amsterdam, The Netherlands; 58 Pro Health Medical, Nederweert, The Netherlands; 59 Department of Molecular Biology, Reinier Haga Medisch Diagnostisch Centrum, Delft, The Netherlands; 60 Laboratory for Medical Microbiology and Immunology, Rijnstate Ziekenhuis, Velp, The Netherlands; 61 Regionaal Laboratorium medische Microbiologie Dordrecht en Gorinchem, Dordrecht, The Netherlands; 62 Royal GD (GD Animal Health), Deventer, The Netherlands; 63 Saltro Diagnostic Center for Primary Care, Utrecht, The Netherlands; 64 Research and Lab Services, Sanquin, National Screening Laboratory, Amsterdam, The Netherlands; 65 Department Medical Microbiology, Slingeland Ziekenhuis Doetinchem, Doetinchem, The Netherlands; 66 Department of Medical Microbiology and Immunology, St. Antonius Ziekenhuis, Utrecht, The Netherlands; 67 Star-shl Medical Diagnostic Center, Rotterdam, the Netherlands; 68 SYNLAB MVZ Leverkusen, Leverkusen, Germany; 69 SYNLAB Heppignies, Heppignies, Belgium; 70 SYNLAB Jena ONCOSCREEN, Jena, Germany; 71 TLR International Laboratories, Ridderkerk, The Netherlands; 72 Department of Diagnostics and Crisis Organization (DCO), Wageningen Bioveterinary Research (WBVR), Wageningen University and Research (WUR), Lelystad, The Netherlands; 73 Department of Medical Microbiology, Ziekenhuis St. Jansdal, Harderwijk, The Netherlands; 74 Department of Medical Microbiology, Zuyderland Medisch Centrum, Heerlen/Sittard-Geleen, The Netherlands

**Keywords:** Microbiology, Molecular biology, Health care

## Abstract

A two-step strategy combining assisted benchmark testing (entry controls) and External Quality Assessments (EQAs) with blinded simulated clinical specimens to enhance and maintain the quality of nucleic acid amplification testing was developed. This strategy was successfully applied to 71 diagnostic laboratories in The Netherlands when upscaling the national diagnostic capacity during the SARS-CoV-2 pandemic. The availability of benchmark testing in combination with advice for improvement substantially enhanced the quality of the laboratory testing procedures for SARS-CoV-2 detection. The three subsequent EQA rounds demonstrated high quality testing with regard to specificity (99.6% correctly identified) and sensitivity (93.3% correctly identified). Even with the implementation of novel assays, changing workflows using diverse equipment and a high degree of assay heterogeneity, the overall high quality was maintained using this two-step strategy. We show that in contrast to the limited value of Cq value for absolute proxies of viral load, these Cq values can, in combination with metadata on strategies and techniques, provide valuable information for laboratories to improve their procedures. In conclusion, our two-step strategy (preparation phase followed by a series of EQAs) is a rapid and flexible system capable of scaling, improving, and maintaining high quality diagnostics even in a rapidly evolving (e.g. pandemic) situation.

## Introduction

High quality pathogen detection systems, with both high sensitivity and specificity, are of paramount importance for public health and individual patient diagnostics^1–3^. In The Netherlands, diagnostic laboratories have the option to choose their own experimental workflows in contrast to many other countries where one or only a few central testing facilities for the whole country are used (e.g. Denmark^4^) or a single workflow type is implemented in multiple decentralized laboratories (e.g. USA^5^). At the start of the SARS-CoV-2 pandemic, no laboratory diagnostic tests for specific SARS-CoV-2 detection were available. Various initiatives were taken to develop specific SARS-CoV-2 tests, including ours at the national reference laboratories for public health action in emerging situations (Dutch National Institute for Public Health and the Environment (RIVM) and Erasmus Medical Centre (Erasmus MC))^1,6,7^. We were involved in the validation of real-time reverse transcription PCR (rRT-PCR) assays for the detection of the novel SARS-CoV-2 virus^1^. This initial assay was based on limited genomic information and developed by Corman et al.^1^ and implemented for Dutch national SARS-CoV-2 testing. In an emerging pathogen situation, like SARS-CoV-2, reference and clinical materials of confirmed positive and negative specimens are largely lacking and procedures for at least verification of the assays with standardized controls is needed. A complicating element was the evolution of the virus resulting in potential mismatched primers leading to false-negative results^8–12^. A widely applied method to evaluate the quality of the complete workflows in diagnostic laboratories (from extraction of nucleic acid to specific virus target detection) is through an External Quality Assessment (EQA)^13–16^. If the test results are unsatisfactory, additional in-depth analyses of the individual components of the workflow can be carried out. In addition, sharing detailed (anonymised) information about workflows and procedures from other laboratories might suggest alternatives and possible solutions.

Here, we describe the application of the combination of an initial benchmark testing (entry-control) procedure using simulated clinical specimens, provision of positive control material and confirmatory testing of patient clinical specimens at the reference laboratory, in which feedback and assistance are offered, followed by periodic EQAs for SARS-CoV-2 molecular diagnostic testing using Nucleic Acid Amplification Tests (NAAT) in 71 diagnostic laboratories in The Netherlands in 2020 and 2021. Passing benchmark testing was necessary for a laboratory to be able to start diagnostic testing or high throughput testing for the general population. We demonstrate that the introduction of the benchmark testing phase before an EQA was highly effective and efficient, and resulted in high quality diagnostic testing. An important aspect of this study is the exploration of additional analysis methods of some steps of/in the workflows. We applied Bayesian statistical modelling to estimate the contribution of the choice of target gene on the Cq values and composed a model that incorporates the effect of individual laboratories. These strategies can identify sensitive steps in the workflows and be helpful to uncover valuable information for the laboratories to improve their performance. Furthermore, the abundant information on Cq values resulting from a high number of different workflows at different laboratories for the same viral concentration specimens, in combination with metadata on strategies and techniques, provided valuable information in the use of Cq values as absolute proxy for viral load. We suggest applying the two-stage strategy and the associated analysis strategy as components of diagnostic preparedness plans for a much wider range of (re-) emerging pathogens of public health concern.

## Materials and methods

### Benchmark testing

Blinded simulated clinical specimen panels (benchmark panel) for sensitivity and specificity analyses were prepared and distributed by the RIVM in collaboration with Erasmus MC. Preparation was performed as previously described^17,18^. Briefly, specimens were prepared in Minimal Essential Medium with Hanks’ salts and Hep2 cells to simulate clinical specimens. The panels contained a randomized dilution series of cultured SARS-CoV-2 and specimens with other related or different viruses were included as analytical specificity controls. A detailed description of the composition of the specimens is given in Supplementary Table 1. Initially, SARS-CoV-1 and also SARS-CoV-2 were included as RNA. As soon as they were available, inactivated Dutch SARS-CoV-2 isolates were included to assess the extraction component in the workflows. Laboratories were asked to report test panel results, as well as information about specimen input volume, extraction volume, elution volume, PCR/NAAT-reaction volume, devices and kits/reagents implemented, and target gene (sequences) for their assays. Alongside the benchmark panels, a positive control specimen initially containing SARS-CoV-1, rapidly replaced by SARS-CoV-2 when available, and validated primers and probes and/or their nucleotide sequence^1^ were supplied for implementing laboratory developed tests in the phase when no commercial detection kits were available. Laboratories implementing solely sample-to-result assays were given the option to test a reduced benchmark panel of four specimens to reduce costly and scarce testing cartridges. In addition, the participating laboratories were requested to supply a minimum of five SARS-CoV-2 positive and 10 SARS-CoV-2 negative tested clinical specimens derived from their own COVID-19 diagnostic pipeline for confirmatory testing at the reference laboratories. Together, these procedures were considered an entry benchmark test. In the event the results returned by a laboratory were unsatisfactory, the laboratory could request another benchmark panel after taking corrective actions. In exceptional cases multiple rounds of benchmark testing were performed. Furthermore, advice was offered by the reference laboratories to improve the technical procedures including the handling of the specimen, the execution of the testing methods, and data analysis. A laboratory’s performance was considered satisfactory during the benchmark testing phase when it was able to test the full panel and the confirmation specimens without false-positive or false-negative results. After a laboratory’s performance was satisfactory it had the freedom to implement other SARS-CoV-2 diagnostic assays, so the new test would be cross-referenced to their primary verified workflow. Laboratories were encouraged to request additional benchmark panels and apply for additional confirmatory testing of clinical specimens to verify novel SARS-CoV-2 diagnostic workflows.

### External Quality Assessment

Three rounds of EQA were performed, in November 2020, February 2021, and May 2021. The EQA panels, consisting of 10 specimens each, were produced in similar fashion as the benchmark panels and their components are described in Table 1. Copies of SARS-CoV-2 E-gene RNA per mL were determined by digital droplet PCR as described previously^17,18^. For sensitivity analyses of SARS-CoV-2, the specimens containing 1.28 × 10^3^ and 1.28 × 10^5^ copies of SARS-CoV-2/mL (referred to as SARS2_L and SARS2_H, respectively) are fundamental as these mimic clinical samples most realistically. The specimen with the lowest virus concentration (1.28 × 10^2^ copies; indicated as SARS2_Edu) was included to get insight into the detection limits of the various workflows. The SARS-CoV-1 containing specimen was included to get an insight into both assay specificity and target gene specificity for pathogens highly similar to SARS-CoV-2, especially as primers and probes specific for SARS-Betacoronaviruses (Sarbecoviruses) are being used^1^. This educational specimen and the SARS-CoV-1 containing specimen were not included in the judgment of the performance of a specific assay regarding applicability for diagnostic testing. Before shipping, the prepared panel was validated at the reference laboratories to confirm expected results. Laboratories were also asked to submit the same metadata as for the benchmark testing phase. The performance per workflow was divided over three performance categories based on the number of false-negative results for SARS-CoV-2, false-positive results for SARS-CoV-2 or inconclusive test results for the non-educational specimens: ‘Excellent’ (100% correct), ‘Mediocre’ (maximally one false positive or negative, or up to two inconclusive) and ‘Unsatisfactory’ (more than one false positive or negative and/or more than two inconclusive). An inconclusive or incorrect result can occur from inadequate specimen preparation or processing or not optimal limit of detection of the NAAT. Specifically, an inconclusive result can be the consequence of differences in individual target results of multi-target tests leading to no clear conclusion concerning the pathogen presence in the tested specimen; not negative and not positive.

**Table 1:** Components of the External Quality Assessment (EQA) specimen panel.

Specimen	Copies of SARS-CoV-2 per mL^a^ or Cq value for not-quantified pathogens	Specimens used in EQA round	Strain, source
SARS-CoV-2 (SARS2_H)	1.28 × 10^5^	1, 2, 3	hCoV-19/Netherlands/NoordBrabant_10003/2020, RIVM
SARS-CoV-2 (SARS2_L)^b^	1.28 × 10^3^	1, 2, 3	
SARS-CoV-2 (SARS2_Edu)	1.28 × 10^2^	1, 2, 3	
SARS-CoV-2 Alpha_H	3.39 × 10^5^	2	hCoV-19/Netherlands/NH-RIVM-20432/2020 B.1.1.7 20B/501Y.V1 , RIVM
SARS-CoV-2 Alpha_L	3.39 × 10^4^	3	
SARS-CoV-2 Beta	1.15 × 10^4^	3	hCoV-19/Netherlands/NH-RIVM-10159/2021, RIVM
SARS-CoV-2 Gamma	8.77 × 10^3^	3	
SARS-CoV-1	Cq 28.57 (Sarbeco E-gene)	1, 2, 3	HKU-39849, kindly provided by Bart Haagmans, Erasmus MC
hCoV-NL63	Cq 28.10 (N-gene)	1, 2, 3	Clinical isolate, kindly provided by Lia van der Hoek, Amsterdam University Medical Center
hCoV-229E	Cq 17.22 (N-gene)	1, 2	ATCC VR-740, ATCC, Manassas, Virginia
hCoV-OC43	Cq 27.77 (N-gene)	1, 2	ATCC VR-1558, ATCC, Manassas, Virginia
Influenza A (H3N2)	Cq 22.76 (M-gene)	1	A/Netherlands/10,078/2020, RIVM
No virus control	n/a	1,2,3	n/a

^a^Copies of E-gene determined by digital droplet PCR as described by Wolters et al., 2020 and Sluimer et al., 2021^17,18^.

^b^Specimen in duplicate in panel.

### Statistical analyses

Statistical analyses were based on a Bayesian model using R (version 4.2.2) and Rstan (R package version 2.21.7) where the measured Cq-value $Cq_{j}$ was assumed to be linearly dependent on the true Cq-value $\mu$. Errors were assumed normally distributed with standard deviation $\sigma$, so that for data point $j$ we have:

1 $$Cq_{j} \sim N\left( {\mu_{j} , \sigma } \right)$$

The Cq-value $\mu_{j}$ is modelled as a sum of components:

2 $$\mu_{j} = \mu_{0} + \mu_{d\left[ j \right]}^{{{\text{dilution}}}} + \mu_{t\left[ j \right]}^{{{\text{target}}}} + \mu_{l\left[ j \right]}^{{{\text{laboratory}}}}$$

The component $\mu_{0}$ is the baseline Cq-value in the specimen labelled as ‘SARS2_H’ containing 1.28 × 10^5^ copies of SARS-CoV-2 per mL, with prior value set to $\mu_{0} \sim N\left( {30,3} \right)$. The component $\mu_{d\left[ j \right]}^{{{\text{dilution}}}}$ is the contribution of the dilution factor at dilution $d\left[ j \right]$ of data point $j$ (which takes values 0 = ’ SARS2_H’, 1 = ’ SARS2_L’, and 2 = ’ SARS2_Edu’). The dilution labelled ‘SARS2_H’ (lowest dilution factor; containing 1.28 × 10^5^ copies of SARS-CoV-2 per mL), is defined as the baseline Cq-value contribution to the dilution-specific term of the model, hence we set $\mu_{0}^{{{\text{dilution}}}} = 0$. For the other dilutions labelled with ‘SARS2_L’ (medium dilution factor; containing 1.28 × 10^3^ copies of SARS-CoV-2 per mL) and ‘SARS2_Edu’ (highest dilution factor; containing 1.28 × 10^2^ copies of SARS-CoV-2 per mL) we expect a correction of respectively $2 \times \log \left( {10} \right)/{\text{log}}\left( 2 \right)$ and 3$\times \log \left( {10} \right)/{\text{log}}\left( 2 \right)$, since we have 2 and 3 log_10_ decreases, and theoretically each halving of the number of genomic copies increases the Cq-value by one. Hence we set priors $\mu_{1}^{{{\text{dilution}}}} \sim N\left( {2\frac{{\log \left( {10} \right)}}{\log \left( 2 \right)}, 0.5} \right)$ and $\mu_{2}^{{{\text{dilution}}}} \sim N\left( {3\frac{{\log \left( {10} \right)}}{\log \left( 2 \right)}, 0.5} \right)$.

The components $\mu_{t\left[ j \right]}^{{{\text{target}}}}$ and $\mu_{l\left[ j \right]}^{{{\text{laboratory}}}}$ are the gene-target and laboratory-specific contributions to the Cq-value. We model those as random effects, i.e. the values they take are assumed to stem from a common distribution:

3 $$\mu_{t\left[ j \right]}^{{{\text{target}}}} \sim N\left( {0,\sigma^{{{\text{target}}}} } \right) {\text{and }} \mu_{l\left[ j \right]}^{{{\text{laboratory}}}} \sim N\left( {0,\sigma^{{{\text{laboratory}}}} } \right)$$

The parameters $\sigma^{{{\text{target}}}}$ and $\sigma^{{{\text{laboratory}}}}$ measure how similar gene-target and laboratory specific Cq-value contributions are. Those are also estimated from the data. We set priors that encode our belief that more than two log_10_ units difference is unlikely:

4 $$\sigma^{{{\text{target}}}} \sim N\left( {0,2} \right)$$

5 $$\sigma^{{{\text{laboratory}}}} \sim N\left( {0,2} \right)$$

Additionally, we enforce sum-to-zero constraints to $\mu_{t\left[ j \right]}^{{{\text{target}}}}$ and $\mu_{l\left[ j \right]}^{{{\text{laboratory}}}}$.

Results that were marked ‘no detection’ were treated differently. For those values the Cq-value was not reported, because no Cq value was generated at all or it is above some Cq threshold. This assessment by the laboratory is unknown to us and could vary between gene-target and dilution. As a substitute we recorded the highest Cq-value found for each combination of ‘dilution’ and ‘gene target’, and used this value (denoted $c\left[ j \right]$) as the censoring level of sample $j$. The censoring is then implemented by using not the probability density function (PDF) for modelling Eq. (1), but the cumulative complementary PDF. This models that Cq-value of non-detects in SARS-CoV-2 containing specimens is somewhere above the censoring level $c\left[ j \right]$, in the tail of the normal distribution Eq. (1).

## Results

### Supporting laboratories to validate and improve their SARS-CoV-2 testing, the benchmark test

**Figure 1:**
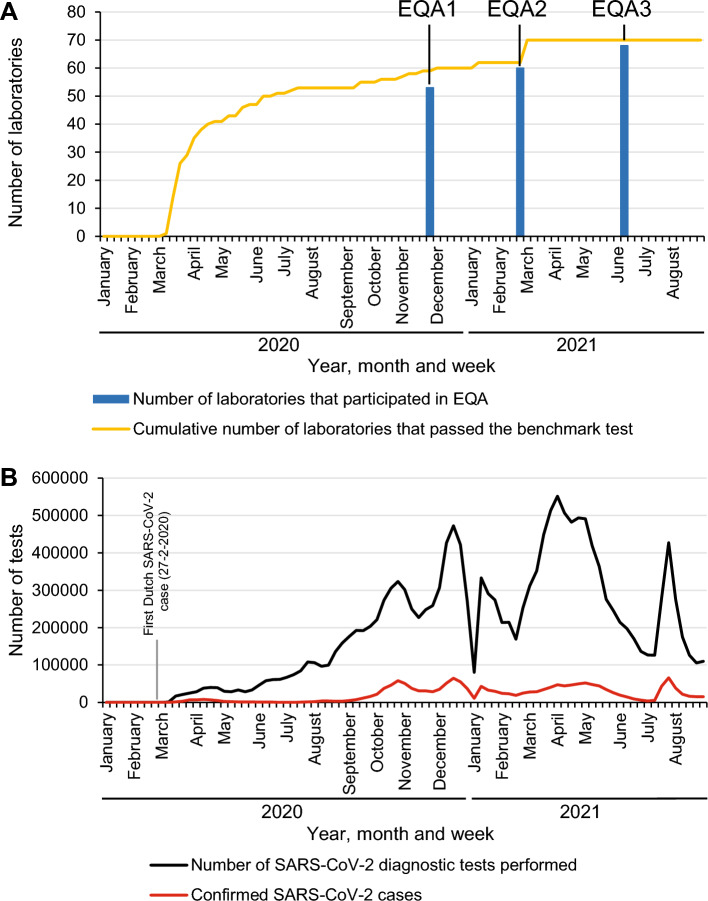
Preparation of diagnostic laboratories for molecular testing of/on SARS-CoV-2 infections in The Netherlands. Already before the first confirmed Dutch SARS-CoV-2 case (February 27th, 2020), the RIVM and Erasmus MC validated and implemented a SARS-CoV-2 RT-PCR test designed by German colleagues^1^. (**A**) During the benchmark testing phase an increasing number of commercial assays became available and were applied in an expanding range of diagnostic laboratories. The benchmark phase was followed by three External Quality Assessment rounds (EQA1-EQA3). (**B**) National data on number of persons tested for SARS-Cov-2 and on number of notified cases were obtained from RIVM data^36^.

As part of the response to the spread of the novel SARS-CoV-2 virus, the reference laboratories assessed and helped to improve the quality of newly introduced workflows for the testing of SARS-CoV-2 in diagnostic laboratories during the early stages of the pandemic. The procedure consisted of two stages, a benchmark testing phase, consisting of the combination of a benchmark panel and a series of confirmation samples, and three rounds of confirmatory EQAs (Fig. 1).

An important aspect of this arrangement was the support offered by the reference laboratories, to assist in the introduction of the workflows and subsequent evaluation thereof. In the benchmark phase multiple technical issues were encountered by some of the laboratories based on the results of the benchmark panel and the confirmation samples that were sent to RIVM. Sensitivity issues were experienced by 15/71 laboratories (21.1%). Also, specificity issues were identified, as 2/71 laboratories (2.8%) were unable to differentiate between SARS-CoV-2 and other (seasonal) coronaviruses. In both cases, RNA isolation and/or amplification techniques were adjusted or substituted which solved the issues. One manufacturer was contacted to improve the performance of three of their kits since laboratories using these kits were experiencing both specificity and sensitivity issues. Contamination issues either during inter-facility specimen transport within the testing laboratory or during testing were experienced in 9/71 laboratories (12.7%). Overall, 56/71 laboratories (78.9%) immediately reached the ‘Excellent’ score whereas 15/71 laboratories (21.1%) needed to implement several adaptations to reach the desired quality level confirmed by testing and passing with another panel. The type of adjustments ranged from fine-tuning their workflow by changing the volumes used during RNA amplification to changing the RNA isolation and/or RNA amplification technique entirely before performance became ‘Excellent’ and passing the benchmark phase.

### Performance of diagnostic laboratories over three EQA rounds

**Figure 2:**
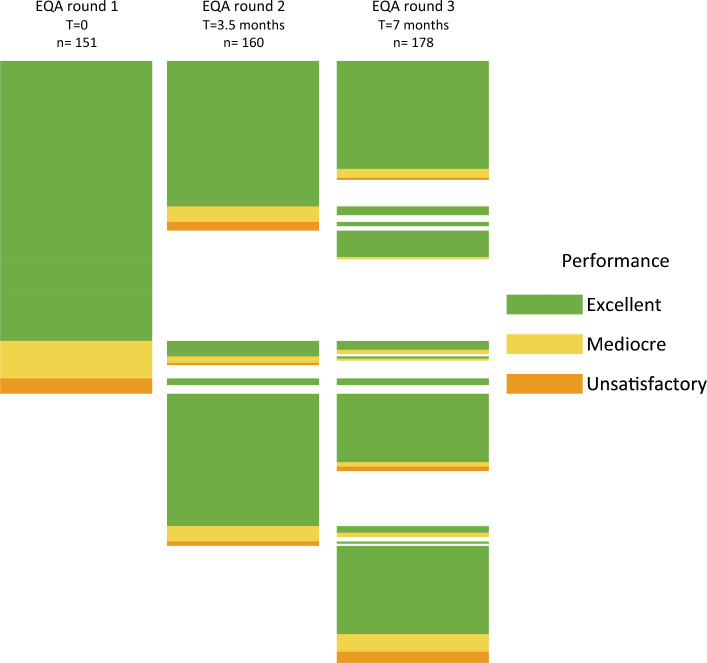
Performance of SARS-CoV-2 diagnostic workflows over three External Quality Assessment (EQA) rounds. Identical workflows used in subsequent EQA rounds are displayed as bars at the same height. Unique workflows are displayed as a bar with no bar to their left and/or right. In total, cumulated over all three EQA rounds 413 excellent performances (127 in EQA round 1, 136 in EQA round 2 and 150 in EQA round 3), 54 mediocre performances (17 in EQA round 1, 17 in EQA round 2 and 20 in EQA round 3), and 22 unsatisfactory performances (7 in EQA round 1, 7 in EQA round 2 and 8 in EQA round 3) were reported. Full details about the workflows used in each EQA round can be found in Supplemental Figs. 4, 5 and 6.

After successfully passing the benchmark test, laboratories took part in up to three EQA rounds which were performed over the course of 7 months. Some laboratories were added to the SARS-CoV-2 testing laboratory network and started and finished the benchmark test after already one or two EQA rounds were completed and therefore could not partake in all three EQA rounds. Other laboratories did not submit data for all EQA rounds despite finishing the benchmark test. In total 53 laboratories participated in EQA1, 60 in EQA2 and 68 in EQA3. The composition of the EQA panels was adapted each round to reflect the occurrence of novel SARS-CoV-2 variants of concern. A schematic overview of the performance of all individual 277 workflows submitted by the 71 laboratories spread over the three EQA rounds is given in Fig. 2.

Many laboratories submitted datasets of multiple workflows which culminated to a total of 489 data sets. The composition of the various workflows was subject to considerable change over time (Supplementary Figs. 4, 5 and 6). An overview of the various target genes applied by the laboratories is given in Supplementary Fig. 1A. Some workflows were deployed in all three EQA rounds while others were used only in one or two rounds (Fig. 2). Remarkably, the overall performance of the workflows did not improve in subsequent rounds. The quality of assays was consistent over the three EQA rounds with approximately 85% of the implemented assays having a 100% score (performance category ‘Excellent’) (Fig. 2). A cumulative overview of the performance on all tested specimens is given in Table 2.

**Table 2:** Overview of the SARS-CoV-2 assay performances over the three External Quality Assessment (EQA) rounds.

Specimen	Number of SARS-CoV-2 assays performed^a^	Correct result (%)	Incorrect result (%)	Inconclusive result (%)
**Sensitivity**				
SARS-CoV-2 (SARS2_H)	465	464 (99.8%)	1 (0.2%)	0
SARS-CoV-2 (SARS2_L)^b^	943	849 (90.0%)	64 (6.8%)	30 (3.2%)
SARS-CoV-2 (SARS2_Edu)^c^	489	252 (51.5%)	196 (40.1%)	41 (8.4%)
SARS-CoV-2 Alpha_H	160	159 (99.4%)	1 (0.6%)	0
SARS-CoV-2 Alpha_L	178	177 (99.4%)	1 (0.6%)	0
SARS-CoV-2 Beta	168	168 (100%)	0	0
SARS-CoV-2 Gamma	168	168 (100%)	0	0
No virus	454	449 (98.9%)	1 (0.2%)	4 (0.9%)
**Specificity**				
SARS-CoV-1^d^	454	212 (46.7%)	199 (43.8%)	43 (9.5%)
hCoV-NL63	489	488 (99.8%)	0	1 (0.2%)
hCoV-229E	286	285 (99.7%)	0	1 (0.35%)
hCoV-OC43	286	286 (100%)	0	0
Influenza A(H3N2)	140	140 (100%)	0	0

^a^The numbers of performed tests are not the same for each specimen as several laboratories did not test all materials. Many laboratories performed testing (of a part of the specimens) on multiple platforms. Not all specimens were included in all three EQA panels.

^b^Panels contained this specimen as a duplicate.

^c^Educational specimen; virus amount below range required for calling positive in screening settings.

^d^Educational specimen.

**Figure 3:**
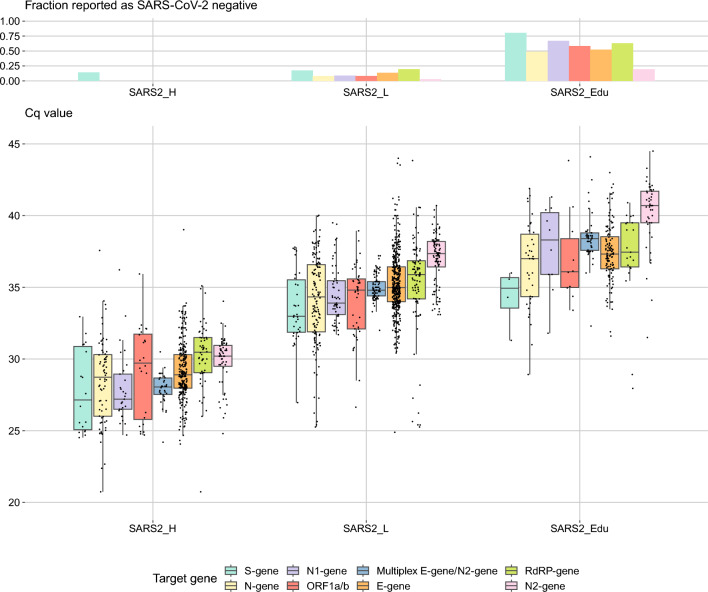
Reported Cq values and the fraction of specimens reported negative for the three SARS-CoV-2 virus concentrations: SARS2_H (1.28 × 10^5^ copies of E-gene/mL); SARS2_L (1.28 × 10^3^ copies of E-gene/mL); SARS2_Edu (1.28 × 10^2^ copies of E-gene/mL). The results are further subdivided per target gene. Target genes utilized less than 10 times are not included. Boxplot hinges represent the 25th and 75th percentile, the middle vertical line is the median, and vertical lines extend to the smallest and largest values no further than 1.5 times the interquartile range from the hinges (values beyond this point can be considered outliers).

As expected, a virus concentration as low as 1.28 × 10^2^ digital copies of E-gene/mL (the educational specimen SARS2_Edu) is a challenge for multiple workflows and resulted in a high proportion (40.1%) of false-negative test results. The various SARS-CoV-2 variants were detected with high accuracy (specificity 99.7%). The specificity of the testing procedures was high, 99.6% of the non-SARS-CoV-2 containing specimens were not mistaken for SARS-CoV-2 except for SARS-CoV-1 which was included in the panels as an educational specimen. Most workflows (53.3%) failed to distinguish SARS-CoV-2 from the closely related SARS-CoV-1 resulting in false-positive results because some workflows solely implemented the E-gene based primers as described by Corman et al.^1^, which cannot discriminate between the two pathogens. However, due to the absence of circulating SARS-CoV-1 since its elimination in 2003, this was not considered a problem. Remarkably, newly developed and implemented assays showed the same high level of quality as pre-existing ones (Fig. 2). Overall, the quality of the implemented workflows was high and stable over time during the study period in which new Variants of Concern emerged. The spread of the reported Cq values by the laboratories over the three sensitivity SARS-CoV-2 specimens is visualized in Fig. 3, in which a subdivision over the target genes is given. Whereas the data for most target genes are produced from multiple assays, the Cq values from the multiplex E-gene/N2-gene are all derived from a single type of cartridge-based sample-to-result assay (Cepheid, Xpert® Xpress SARS-CoV-2/Flu/RSV assay). We observed the least spread of Cq values with this last assay (24.2–30.5), whereas the spread overall for the other assays was 18.11–39.02 for specimen SARS2_H. For each of the targets a lower concentration resulted in a higher Cq value (connectors between specimens for individual workflows not shown in Fig. 3).

The reported Cq values are systematically higher than theoretically expected based on the dilutions (Supplementary Fig. 2). Supplementary Fig. 3 shows the predicted versus the reported Cq values of the individual target genes when plotted against each other. These data demonstrate that there is no strict linear correlation between Cq value and viral concentration in the studied concentration range and that this is independent of the choice of target gene.

### Quantification of the contribution of some parameters to workflow performance

**Figure 4:**
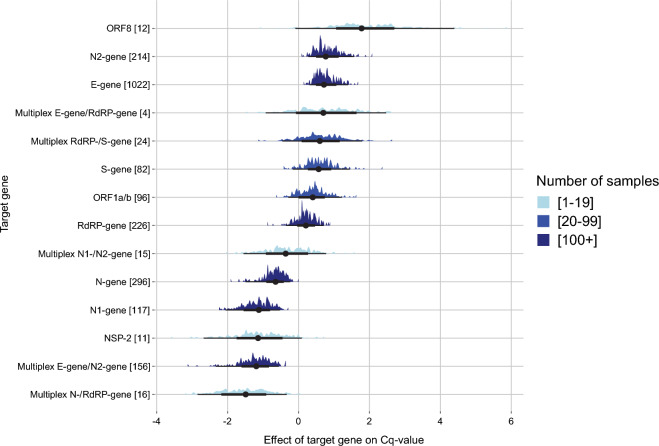
Visual representation of the effect of ‘Target gene’ on the reported Cq values as probability distributions. The X-axis depicts the spread in predicted Cq value per target gene (Y-axis) relative to the mean Cq value of all target genes combined. The median (black dot) and 50% and 95% distribution intervals (black lines) are indicated per target gene. The total number of datapoints for each target gene is shown in brackets. The different sizes of datasets are displayed using light blue (range < 20), blue (range 20–99) and dark blue (100+).

To infer the quantitative effect of the choice of target genes on the assay read-out parameter Cq, we applied Bayesian statistic modelling. This method estimates the likelihood of a Cq value as a distribution while correcting for confounding factors (for details see “Methods” section). To determine what the effect of the chosen target genes on the reported Cq values is, we modelled this effect for the SARS-CoV-2 specimens SARS2_H, SARS2_L and SARS2_Edu. Figure 4 shows the (mean) effect of target gene selection on derived Cq values for an assay. The predicted range is a parameter of the number of data points and that over all individual target genes, the mean values are distributed over a range of about 3.5 Cq values. Such data could be useful for selecting a new target gene for an assay if necessary. Similarly as for the target genes, we modelled the contribution of ‘Laboratory’ on the reported Cq values (for details see Methods section). This ‘Laboratory’ effect on the predicted Cq values for all types of assays ranged from -2.3 to 3.2 from the mean (Supplementary Fig. 1, panel B). The laboratories CS and CT occupy a relatively separate position which can possibly be attributed to the relative high number of specimens incorrectly reported as SARS-CoV-2 negative due to sensitivity issues. Thus, even when adjusting for the ‘Laboratory effect’ the difference in Cq values between laboratories for same concentration specimens remained considerable, further illustrating that taking Cq values as absolute proxy for viral load between laboratories and assays has limited value.

## Discussion

This study describes a successful strategy for assessment, increasing and maintaining the quality of molecular diagnostics for SARS-CoV-2 in a very heterogeneous laboratory landscape by combining a benchmark testing phase and an EQA phase. These establishment and evaluation procedures were of great importance for setting up diagnostic testing facilities throughout The Netherlands in an early phase of the pandemic.

The Netherlands chose to implement decentralised testing with a wide variety of SARS-CoV-2 assays, the same approach that was also chosen for The Netherlands during the 2009 influenza pandemic^16^. This strategy has challenges as it is potentially difficult to maintain a homogenous high-quality level in a heterogenous testing landscape. This issue can be resolved by a well-designed test-implementation system with regular EQA and inter-laboratory comparisons as shown in this study. Importantly, a laboratory network implementing a multitude of assays essentially reduces the risk of collapse of the complete testing landscape (don’t put all of your eggs in one basket)^19^. During the COVID-19 pandemic, multiple issues were encountered including manufacturing problems, contamination of primers/probes^20^ and drop-outs because of genomic mutations in target genes^8–12,21^. In contrast to The Netherlands, the USA took the approach of decentralised testing with one assay type, similar to what they did during the 2009 influenza pandemic^22,23^. Although this method generally allows for quick and relatively simple upscaling of diagnostic capacity, when this strategy was implemented for SARS-CoV-2 in 2020 in the USA it had its challenges, namely contamination of primers/probes with synthetic template and improper primer/probe design^20,24–26^ which impaired the testing system. The CDC had a similar experience when implementing an mpox assay in their laboratory network in 2022^27,28^. While we acknowledge that this topic is too complex to be discussed thoroughly in our paper, we feel it is worth briefly mentioning in this discussion as a way of starting or adding to pandemic preparedness systems discourse.

An important characteristic of the strategy of implementing heterogenous assays in The Netherlands was the presence of a ‘preparation phase’ (benchmark testing). In this phase laboratories could already make use of readily available blinded panels of simulated clinical specimens containing SARS-CoV-2 and other viruses during the early stage of the pandemic, and in addition receive advice and support from the reference laboratories. We observed that during this preparation phase the performance of several laboratories improved considerably, resulting in high quality testing in these laboratories and meeting set requirements for inclusion in the list of qualified SARS-CoV-2 diagnostics laboratories^29^. Most issues were found in high volume laboratories that previously did not perform diagnostics on human-derived specimens, which included veterinary laboratories and newly set up laboratories specific for SARS-CoV-2 testing, among others.

After finishing the benchmark phase, 84.5% of all submitted workflows performed up to the desired level in the subsequent individual EQA rounds. This is remarkable, as according to the benchmark inclusion criteria, all workflows were expected to perform ‘Excellent’ in the EQA rounds. It is possible that not all new workflows implemented in laboratories were pre-tested in the benchmark phase. However, our data does not provide a clear explanation for this observation.

The first published SARS-CoV-2 EQA was performed, primarily focused on frontline diagnostic laboratories, in April/May of 2020^30^. In this first EQA, 365 of 406 laboratories from 36 countries submitted 521 datasets. All core samples from the EQA were correctly reported by 86.3% of participating laboratories and 83.1% of the datasets^30^, similar to our study. In another early SARS-CoV-2 EQA (which focused more on “expert” and reference laboratories, rather than frontline diagnostic laboratories) among 68 diagnostic laboratories spread over 35 European countries^14^, the test performances were of significantly lower quality than in our study (39.7% versus 84.5% of workflows scored all core specimens correct). The percentage of false positives or negatives in our study were 3.2% false negative, 0.1% false positive, whereas Fischer and colleagues found 8.6% false negative and 1.1% false positive results in their European study^14^. The Fischer et al. EQA was performed in June and July 2020 while our EQAs started in December that same year^14^. As laboratories had more time to set up their assays before the start of our study compared to the laboratories partaking in the Fischer et al. study^14^, the difference in diagnostic quality between the two studies might be partly due to more experience in COVID-19 diagnostics at the partaking laboratories. A major difference with our study is that the Fischer et al. or Matheeussen et al. study did not involve a benchmark testing procedure in advance of the EQAs^14,30^. The benchmark testing phase in our study started as early as March 2020 and could be considered an individual EQA with strict targets to be met by the laboratories. Nevertheless, this actually shows the benefit of our systematically applied entry benchmark testing approach that was (largely) lacking in other approaches. Based on our results, we expect that the availability of blinded testing panels to validate assays during implementation and compare performance with that of other laboratories, in combination with technical support, could improve the quality of the diagnostic testing performance in laboratories elsewhere. It is of note that this strategy is widely applicable and can cover other (novel) pathogens as well.

Other national SARS-CoV-2 diagnostic testing EQAs were performed and documented in Japan (94.1% correct reporting)^31^, South Korea (93.2% correct reporting)^15^, and Austria (93% correct reporting)^32^, with mostly similar results. Comparing these EQAs, or the original EQA from Matheeussen et al.^30^, with our EQA program is challenging, as sample quantification and preparation were done differently (in our study, using Minimal Essential Medium with Hanks’ salts and Hep2 cells instead of transport medium for sample preparation, varying methods for virus concentration determination, and using inactivated SARS-CoV-2 virus instead of RNA or pseudovirus constructs). It is of additional value when, in addition to the test results of the panel specimens, detailed information about the technical and procedural aspects, the so-called metadata, are shared with the organizer of an EQA. Communicating an overview of these anonymized and aggregated data, which cannot be collected within individual laboratories, among all EQA participants might be informative for an individual laboratory to compare its own quality level with its peers and especially, for getting suggestions for alternatives in case of suboptimal performance. In this report, we have taken this analysis a step further and demonstrate the possibility to get insights into specific aspects of the workflows. Such information can hint at steps in the procedures that are critical and it provides a quantitative estimate of its impact. Here we show a comparison of the consequences of the various target genes used by the laboratories on the workflows. This provided a direct comparison of target genes and suggests validated alternatives in case a gene target is no longer available because of mutations. Such analyses can also be performed for other elements of the workflows or even as a comparison between laboratories as we demonstrate.

A much debated topic is the use of Cq values as a measure for the absolute amount of virus in a clinical specimen. Differences in the amount of mRNA for protein production between the various targeted genes besides the presence of viral genomic and subgenomic RNA and differences in stability of the various RNAs will influence the amount of substrate for the RT-PCR reaction^33,34^. This is supposedly also in part reflected in the results of our analyses of the influence of target genes on Cq values. These factors limit a direct use of Cq values for virus concentration, apart from the difficulty to collect a specimen from a host in a reproducible way. Even standardised sample-to-result assays, which excludes some technical variation by making use of cartridges, showed substantial spread in Cq values in our study. Therefore, Cq values without calibration to international standards cannot be used to determine the amount of virus reliably and can at most provide a rough estimate, as for all workflows analyzed a decrease in concentration resulted in an increase in Cq value. The N2 target region seemed to be most sensitive target with the highest percentage of workflows with a positive result for the SARS2_Edu specimen, although Cq values were the highest compared to the other targets for this specimen. Differential generation of subgenomic mRNA’s^35^ and differences in reaction efficacy at low target concentration in a specimen—we show amplification is not exponential anymore (Supplementary Fig. 2 and 3)—could explain this phenomenon.

We consider the system of initial provision of validated primers and probes and protocols for laboratory developed tests and subsequent entry benchmark testing as an excellent way to develop and improve molecular diagnostic testing of pathogens in emerging situations requiring rapid availability of validated assays and high testing capacity. Technical and logistic assistance from a public health institute and/or expert laboratory is an important component. As this report demonstrates, the program is highly flexible and fast, allowing laboratories to design or purchase their own preferred assays and workflows while verifying and maintaining high quality testing. The addition of timely followup EQA rounds are necessary to maintain the overall quality of the diagnostic network. The collection and exchange of metadata is a valuable component and sophisticated statistical analyses can provide informative insight to the laboratories into components of their workflows. Importantly, the strategies here described are applicable to other pathogens as well and can be of great value in improving preparedness for novel pathogen detection and contribute to the advance of public health in a continuously developing and changing diagnostic field.

### Supplementary Information

Supplementary Information 1.

Supplementary Information 2.

## Data Availability

Raw data is available as supplemental file.
