# Supplementary material for: High quality of SARS-CoV-2 molecular diagnostics in a diverse laboratory landscape through supported benchmark testing and External Quality Assessment
Source: Sci Rep. 2024 Jan 16;14:1378. doi: 10.1038/s41598-023-50912-9 (PMC10792020; doi:10.1038/s41598-023-50912-9)
Supplement: Supplementary file 2 — Supplementary Information 2. [file 41598_2023_50912_MOESM2_ESM.pdf]

Supplementary material

Supplementary Table 1. Components of benchmark testing panels 1 – 6 and V1

Panel specimen	Copies of SARS-CoV-2 per mL *	Cq value of SARS-CoV-2 specimen †	Target specific Cq value (non-SARS-CoV-2) †,‡	Included in benchmark panel(s) §	Source
SARS-CoV-2_Bm_1	-	23.3	-	2, 3	BetaCoV/Germany/BavPat1/2020; RNA, provided by Charité - universitätsmedizin Berlin, Berlin
SARS-CoV-2_Bm_H<br>SARS-CoV-2_Bm_L<br>SARS-CoV-2_Bm_Edu ¶	1.28×10^5<br>1.28×10^3<br>1.28×10^2	26.9 - 28.5<br>33.1 - 34.8<br>34.9 - 35.6	-<br>-<br>-	4, 5, 6, V1<br>4, 5, 6 (duplicate), V1<br>4, 5, 6, V1	hCoV-19/Netherlands/NoordBrabant_10003/2020
hCoV-229E	-	-	27.2 - 30.2	1, 2, 3, 4, 5, 6	ATCC, Manassas, Virginia (ATCC VR-740)
hCoV-NL63	-	-	24.1 - 28.1	1, 2, 3, 4, 5, 6	Clinical isolate kindly provided by Lia van der Hoek, Amsterdam University Medical Hospital
hCoV-OC43	-	-	27.3 - 30.8	1, 2, 3, 4, 5, 6	ATCC, Manassas, Virginia (ATCC VR-1558)
Rhinovirus A16	-	-	19.9 - 25.5	1, 2, 3, 4, 5	Clinical isolate, RIVM
Influenza A(H3N2)	-	-	19.8 - 22.8	1, 2, 3, 4, 5, 6	A/Netherlands/10078/2020
Influenza virus B-Victoria_1	-	-	22.3 - 28.3	1, 2, 3, 4	B/Netherlands/2423/2017
Influenza virus B-Victoria_2	-	-	28.8	5	B/Netherlands/10063/2020
SARS-CoV-1 (1.1)<br>SARS-CoV-1 (1.2)<br>SARS-CoV-1 (1.3)<br>SARS-CoV-1 (2.1)<br>SARS-CoV-1 (2.2)	-<br>-<br>-<br>-<br>-	-<br>-<br>-<br>-<br>-	24.2<br>28.2<br>32<br>28.6 - 28.9<br>32.3	1<br>1<br>1<br>2, 3, 5, 6<br>2, 3	HKU-39849; RNA, kindly provided by Bart Haagmans, Erasmus MC, virus RNA in lysis buffer
No virus	-	-	-	1, 2, 3, 4, 5, 6, V1	n/a

* Based on a digital droplet PCR using an RIVM in-house assay.

† Expected Cq values shown in this table are based on RT-qPCR tests performed on the panel samples using the routinely used RIVM in-house assays.

‡ For SARS-CoV-2 and SARS-CoV-1 = E-gene; hCoV-229E, hCoV-NL63, hCoV-OC43 = N-gene; Rhinovirus A16 = 5’non-coding-region; Influenza virus type A = M-gene; Influenza virus type B/Victoria: HA-gene.

§ Panel 1 was accompanied with a SARS-CoV-1 RNA 10-fold dilution series, panels 2 and 3 were accompanied with a SARS-CoV-2 RNA 10-fold dilution series and panels 4, 5 and 6 were accompanied with an inactivated SARS-CoV-2 whole virus 10-fold dilution series. These dilution series were included to provide a- raw estimate of the limit of detection of workflows. Panel V1 was a limited panel with less specimens intended for verification of sample-to-result assays.

¶ This specimen was considered an educational specimen. Laboratories were not required to detect SARS-CoV-2 in this specimen.

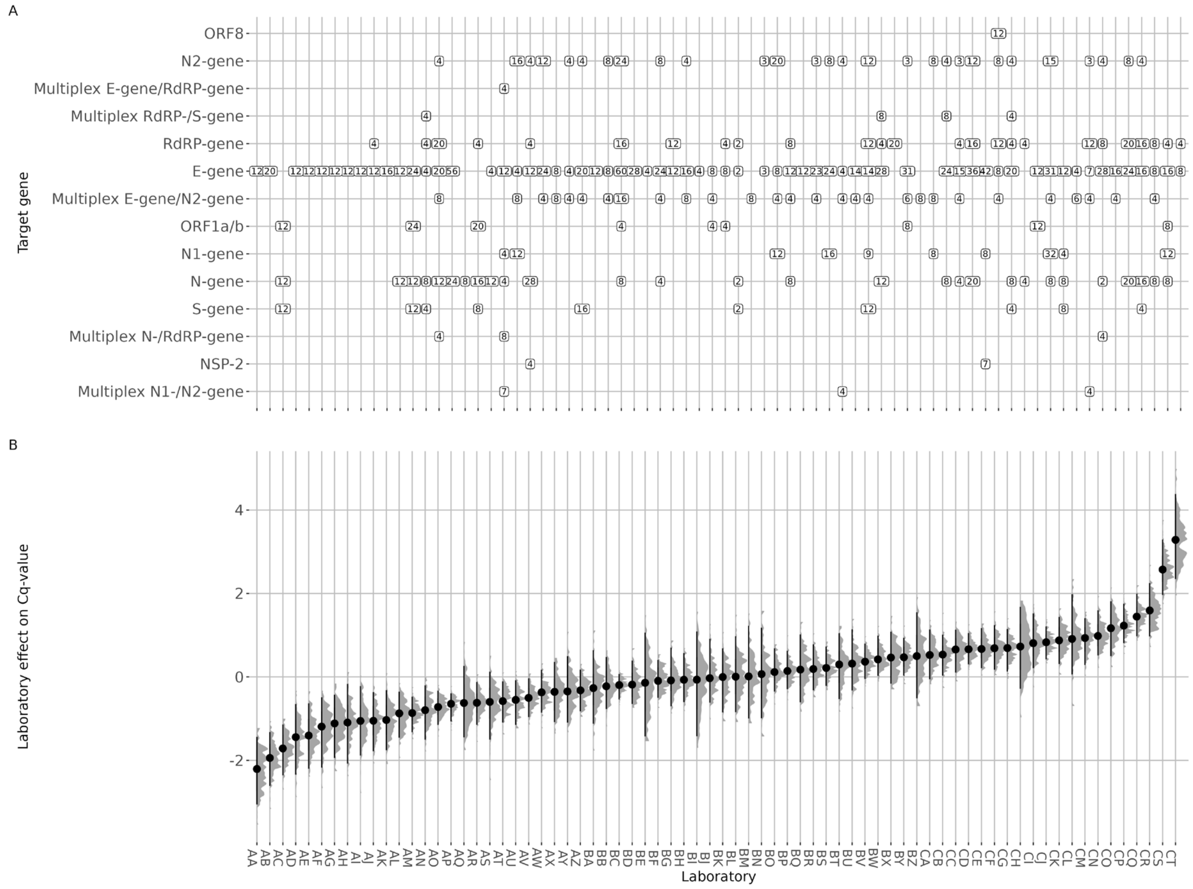

Supplementary Figure 1. Overview of the choice and frequency of use of applied target genes by the laboratories (panel A). Predicted effects of the individual laboratories on their reported Cq values (panel B). Gray areas correspond to the posterior probability density functions (PDF's) for the laboratory dependent part of the model. Thick/thin lines span the 50% and 95% credible interval, the black dots are the medians of the distribution. The laboratories have been anonymized.

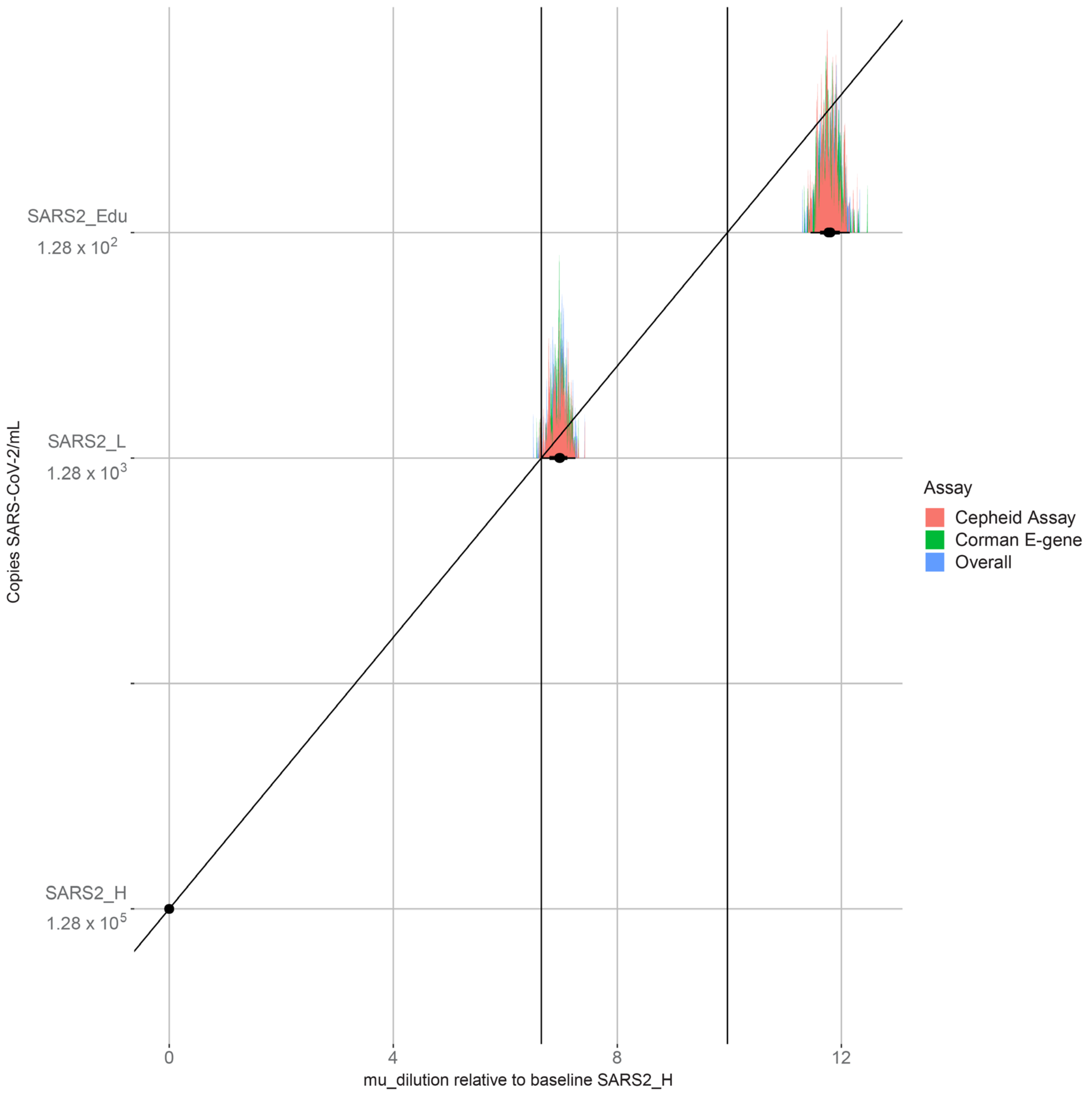

Supplementary Figure 2. Obtained (colored distributions) and expected (vertical black lines) values of μ^{dilution} (i.e. the dilution specific contribution to the Cq-values relative to the SARS2_H specimen). Blue color indicates all assays combined, green is Corman E-gene (2020) and red the Cepheid Assay (Cepheid, Xpert® Xpress SARS-CoV-2/Flu/RSV assay). Results are presented for SARS2_H, SARS2_L and SARS2_Edu specimens. As detailed in the Methods section, SARS2_H is set at zero as a baseline value. Predicted Cq value contributions for the SARS2_L and SARS2_Edu specimens would be located at crossings of the diagonal line with the vertical lines in case of a linear relationship. The mean Cq difference increased by seven cycles for the SARS2_L specimen compared to the SARS2_H specimen which differ by a factor of 100 in (theoretical) viral load. Similarly, the mean Cq value increased by 4.9 for the SARS2_Edu specimen compared to the SARS2_L specimen that differ by a factor of 10 in viral load. These values lie above the theoretical estimated increase in Cq values of 6.6 ($\log_2(100)$) and 3.3 ($\log_2(10)$), respectively. The overlapping distributions indicate that the results are robust with respect to assay.

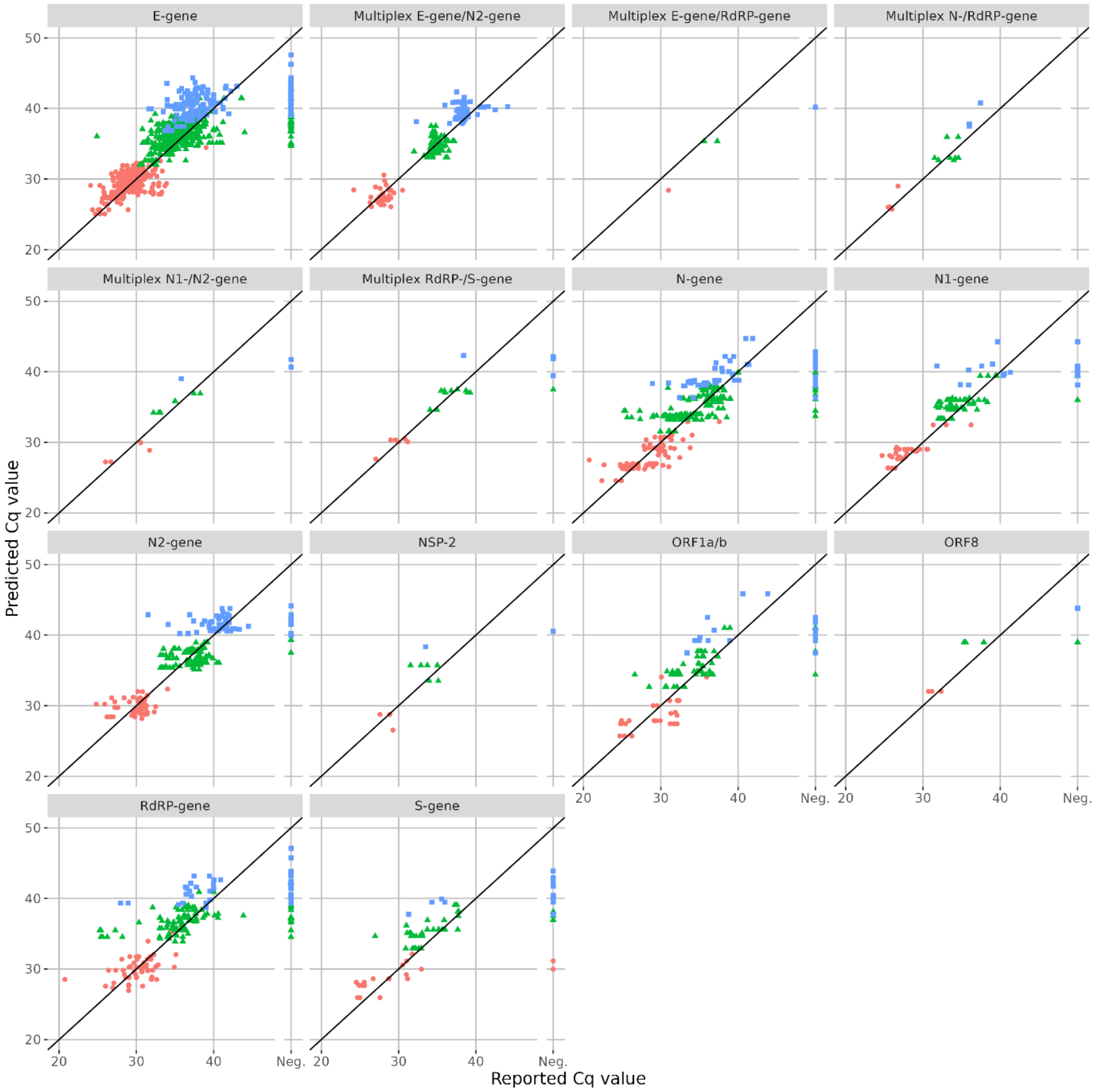

Supplementary Figure 3. Reported and predicted Cq values of all target genes for SARS2_H (red circles), SARS2_L (green triangles) and SARS2_Edu (blue squares) specimens. On the far right of each panel false negative results are shown.

In the following three Supplemental Figures 4, 5 and 6, summarising the great variety in workflows used in the EQA studies, a scoring system for performance in the EQA was used. This scoring is based on the eight core specimens in a panel. SARS-CoV-1 containing, and lowest concentration SARS-CoV-2 educational specimens were excluded from the scoring. Each workflow started with eight points for all correct results. For each of the eight core specimens, for each wrongly determined core specimen (being positive for a specimen containing no SARS-CoV-2 or vice versa) one point was deducted. When a sample was scored with an 'Indeterminate', 'Equivocal' or 'Inconclusive' result, 0.5 points were deducted from the final score of the workflow. For some workflows (e.g. molecular point-of-care test (mPOCT) workflows) an option was given to test only five preselected core specimens to limit costs and number of tests when they were scarce. These workflows were also scored according to a scale from 0 – 8 points. For each wrongly determined specimen (being positive for a specimen containing no SARS-CoV-2 or vice versa) two points were deducted. When a specimen was scored with an "Indeterminate", "Equivocal" or "Inconclusive" result, one point was deducted from the final score of the workflow. For each workflow that reported 'Not tested' panel specimens, one point was deducted for each 'Not tested' specimen. For Figure 2 in the manuscript these scores have been translated in 'Excellent' (100% correct; score of 8), 'Mediocre' (maximally one false positive or negative, or up to two inconclusive; scores 7.5 and 7) and 'Unsatisfactory' (more than one false positive or negative and/or more than two inconclusive; scores lower than 7). Workflows described in Supplementary Figures 4, 5 and 6 that were used to test a random subset of specimens, experimental workflows and workflows belonging to laboratories that eventually did not participate in the national testing strategy were not included in the analyses described in the manuscript.

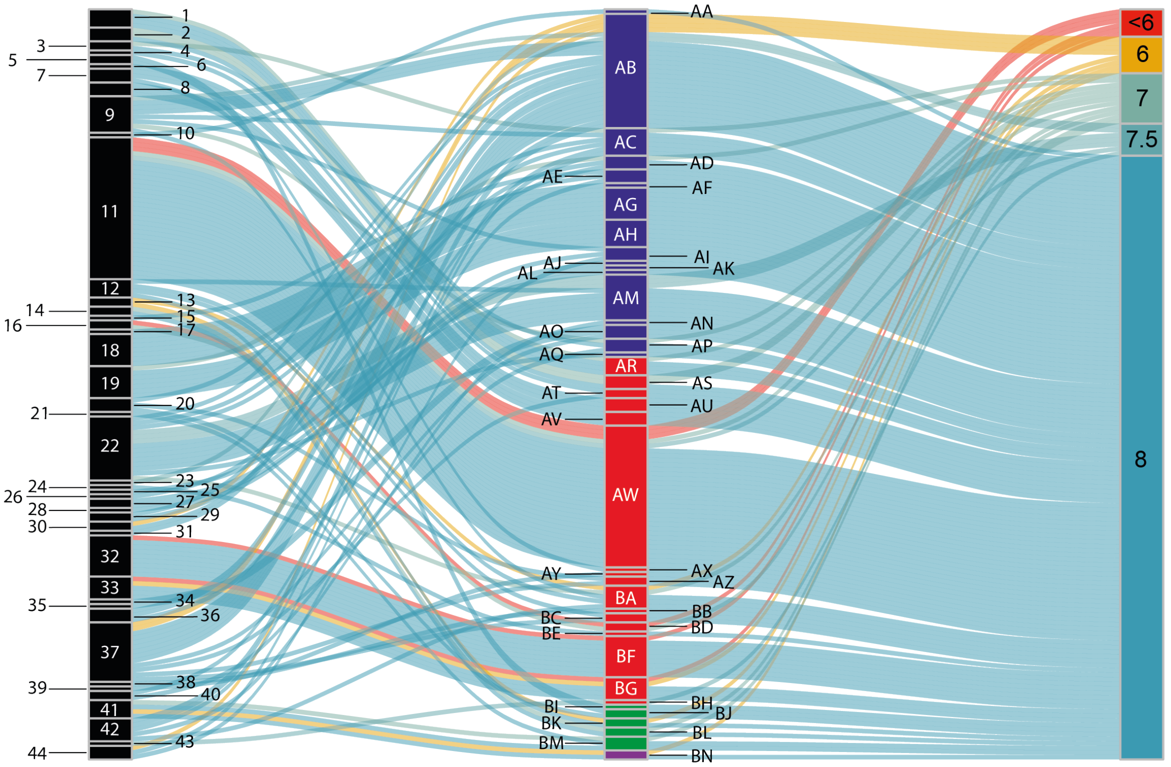

RNA isolation methods	
Abbott, ALINITY m SARS-CoV-2 ASSAY	1
Abbott, Sample Preparation System DNA kit	2
Abclonal, ANDiS Viral Nucleic RNA Auto Extraction & Purification Kit	3
Altona, Altostar Purification kit 1.5	4
Applied Biosystems, MagMAX TM Viral/Pathogen II Nucleic Acid Isolation Kit	5
BD, BD MAX SARS-CoV-2 kit	6
BD, BD MAX ExK TNA-3	7
BioMerieux Diagnostics, Respiratory panel 2.1 plus	8
BioMerieux, NUCLISENS® easyMAG®	9
Biotecon Diagnostics, Magnetic Preparation Kit VI	10
Cepheid, Xpert® Xpress SARS-CoV-2	11
Chemagen, Chemagic Viral DNA/RNA 300 kit H96	12
Clean NA, Viral DNA & RNA SP kit	13
ElitechGROUP, ELITe InGenius SP 200	14
GeneFinder COVID-19 Plus	15
GenMark Dx, Eplex RP2	16
GSD NovaPrime®, IVD RNA Extraction AE1 kit	17
Hologic, Aptima (R) SARS-CoV-2 assay	18
Hologic, Panther Fusion® SARS-CoV-2 assay	19
Life Technologies, MagMax pathogen RNA/DNA kit	20
Magtivio, MagSi-NA Pathogens kit	21
Molg3n, PurePrep Pathogens	22
NeuMoDx™, RNA mastermix strip	23
NeuMoDx™, SARS-CoV-2 Assay	24
Precision System Science Co., E1300 MagDea isolatie chemie	25
Promega, Maxwell RSC Blood DNA kit	26
Qiagen, QIAprep&amp Viral RNA UM Kit	27
Qiagen, QIAstat-Dx Respiratory SARS-CoV-2	28
Qiagen, QIAsymphony DSP Virus/Pathogen Midi Kit	29
Qiagen, QIAsymphony DSP Virus/Pathogen Mini Kit	30
RBCBioscience, MagCore® Viral Nucleic Acid Extraction Kit	31
Roche, Cobas Liat SARS-CoV-2&Influenza A/B	32
Roche, Cobas SARS-CoV-2: Qualitative assay for use on the cobas 6800/8800 Systems	33
Roche, Cobas® 4800 CT/NG Test	34
Roche, Cobas® 4800 Human Papillomavirus (HPV) Test	35
Roche, DNA and Viral NA Large Volume kit	36
Roche, DNA and Viral NA Small Volume kit	37
Roche, MagNA Pure 24 Total NA Isolation Kit	38
Roche, MagNA Pure 96 Total Nucleic Acid Isolation Kit	39
Roche, MagNA Pure LC Total Nucleic Acid Isolation Kit	40
Seegene, STARMag 96x4 Universal Cartridge Kit	41
Siemens, VERSANT Sample Preparation 1.0 Reagents	42
Zeesan, lab-aid Virus RNA extraction kit	43
Unknown	44

PCR/NAAT methods	
**Tests with 1 target gene**	
Biotecon Diagnostics, microproof SARS-CoV-2 Identification Kit	AA
Corman et al. (2020) E-gene Sarbeco specific	AB
Corman et al. (2020) E-gene SARS-CoV-2 specific	AC
Corman et al. (2020) RdRP-gene	AD
E-gene Sarbeco specific (Unknown sequence)	AE
GSD, NovaPrime® SARS-CoV-2 (COVID-19) RT-PCR kit	AF
Hologic, Aptima (R) SARS-CoV-2 assay	AG
Hologic, Panther Fusion® SARS-CoV-2 assay	AH
Lu et al. (2020) N-gene	AI
Qiagen, QIAprep&amp Viral RNA UM Kit	AJ
Qiagen, QIAstat-Dx Respiratory SARS-CoV-2	AK
Qiagen, SARS-CoV-2 N1+N2 Assay Kit	AL
r-Biopharm, RIDAGENE SARS-CoV-2 RUO	AM
Siemens, FTD Sars-CoV-2 assay	AN
TibMolBio, LightMix® Modular Sarbecovirus E-gene, CE	AO
TibMolBio, SARS-CoV-2	AP
Unknown	AQ
**Tests with 2 target genes**	
Abbott, ALINITY m SARS-CoV-2 ASSAY	AR
Abbott, M2000 SARS-CoV-2	AS
Altona Diagnostics, Altona Realstar SARS-CoV-2 RT PCR	AT
BD, SARS-CoV-2 kit	AU
BioMerieux Diagnostics, Respiratory panel 2.1 plus	AV
Cepheid, Xpert® Xpress SARS-CoV-2	AW
Certest, VIASURE SARS-CoV-2 Real Time PCR	AX
Corman et al. (2020) E-gene Sarbeco specific; Corman et al. (2020) E-gene SARS-CoV-2 specific	AY
Corman et al. (2020) E-gene Sarbeco specific; Corman et al. (2020) RdRP-gene	AZ
Corman et al. (2020) E-gene Sarbeco specific; Lu et al. (2020) N1-gene	BA
Corman et al. (2020) E-gene Sarbeco specific; Lu et al. (2020) N-gene	BB
Corman et al. (2020) E-gene SARS-CoV-2 specific; Lu et al. (2020) N1-gene	BC
GenMark Dx, Eplex RP2	BD
NeuMoDx™, SARS-CoV-2 Assay	BE
Roche, Cobas Liat SARS-CoV-2&Influenza A/B	BF
Roche, Cobas SARS-CoV-2: Qualitative assay for use on the cobas 6800/8800 Systems	BG
Xiamen Zeesan Biotech Co., Ltd., SARS-CoV-2 Test Kit (Real-time PCR)	BH
**Tests with 3 target genes**	
Applied Biosystems, TaqPath COVID-19 RT-PCR Kit	BI
Applied Biosystems, TaqPath COVID-19 Multiplex Diagnostic Solution (CE-IVD)	BJ
Chemagic, SARS-CoV-2 RT-q PCR Perkin Elmer	BK
ELITechGroup, GeneFinder COVID 19 PLUS Real Amp Kit	BL
Seegene, Allplex 2019-nCov Assay	BM
**Test with 4 target genes**	
Seegene, Allplex SARS-CoV-2 Assay	BN

Supplementary Figure 4. A flow diagram showing all workflows reported that have tested the EQA panel 1 with extraction method, PCR test, the number of target genes used, and the final score achieved by each workflow. In the alluvial plot PCR tests using one target gene are depicted in blue, PCR tests using two target genes are shown in red, PCR tests using three target genes are shown in green and PCR tests using four target genes are shown in purple. The color of trails per workflow are based on the score obtained for EQA panel 1. All workflows receiving scores below 6 are grouped in <6.

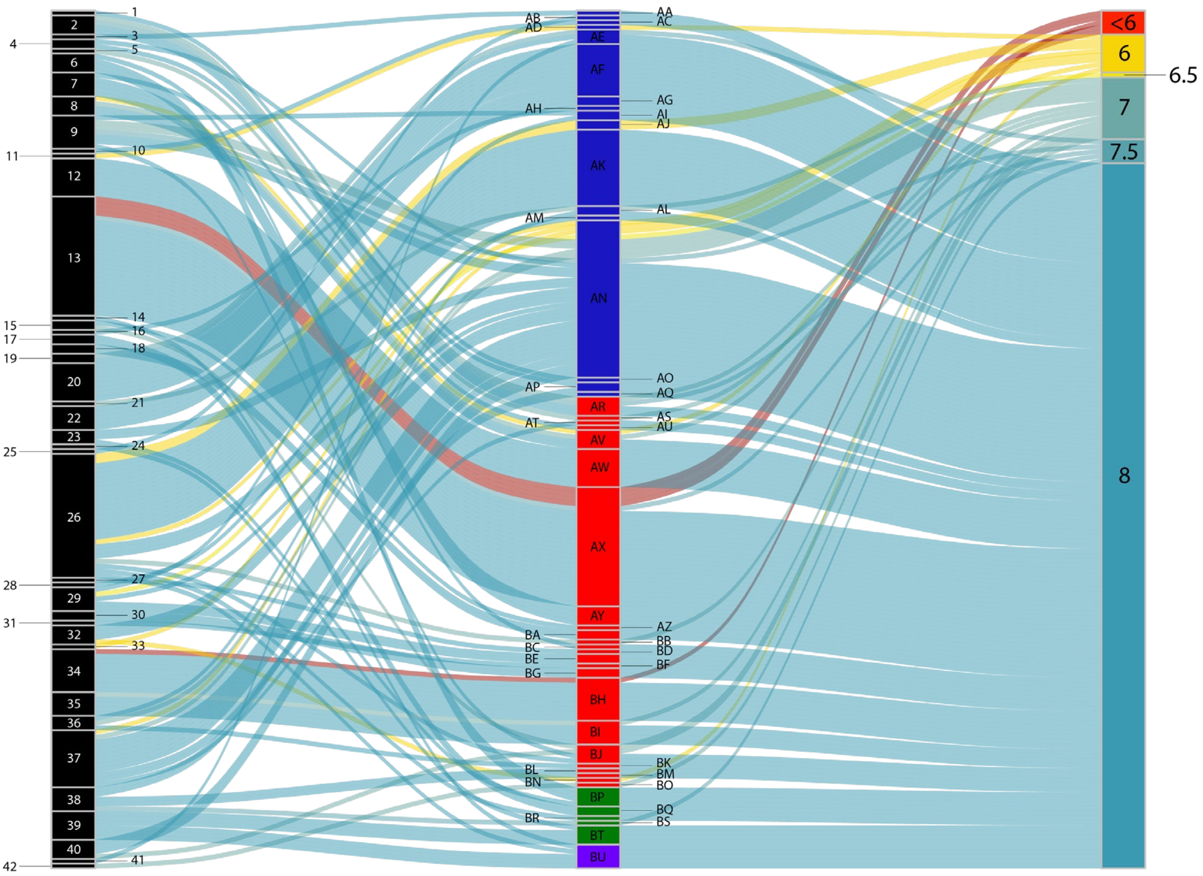

RNA isolation methods	
A&A Biotechnology, MagnifiQ RNA Kit	1
Abbott, ALINITY m SARS-COV-2 ASSAY	2
Abbott, ID NOW™ COVID-19	3
Abclonal, ANDiS Viral Nucleic RNA Auto Extraction & Purification Kit	4
Abott, M2000 SP	5
Applied Biosystems, MagMAX TM Viral/Pathogen II Nucleic Acid Isolation Kit	6
BD, BD MAX ExK TNA-3	7
BioMerieux Diagnostics, Respiratory panel 2.1 plus	8
BioMerieux, NUCLISENS® easyMAG®	9
Biotecon Diagnostics, Magnetic Preparation Kit VI	10
Bosch, Vivalytic CoV Viral SARS-CoV-2 test	11
Cepheid, Xpert® Xpress SARS-CoV-2	12
Cepheid, Xpert® Xpress SARS-CoV-2/Flu/RSV	13
Chemagen, Chemagic Viral DNA/RNA 300 kit H96	14
Chemagic, Viral DNA/RNA Kit	15
Diagenode Diagnostics, MagDEA Dx cartridges	16
ElitechGROUP, ELITe InGenius SP 200	17
GenMarkDx, ePlex® Respiratory Pathogen Panel 2	18
Goldstandard Diagnostics, NovaPrime RNA Extraction AE1	19
Hologic, Aptima® SARS-CoV-2 assay	20
Hologic, Panther Fusion® Open Access™	21
Hologic, Panther Fusion® SARS-CoV-2 assay	22
Life Technologies, MagMax pathogen RNA/DNA kit	23
Magtivio, MagSi-NA Pathogens	24
MGI, MGIEasy Nucleic Acid Isolation Kit	25
Molgen, PurePrep Pathogens	26
Procomcure Biotech, SphaeraMag DNA/RNA Isolation Kit - prefilled96	27
Promega, Maxwell RSC Blood DNA kit	28
QIAGEN, NeuMoDx™ SARS-CoV-2 Assay	29
QIAGEN, QIAprep& Viral RNA UM Kit	30
QIAGEN, QIAstat-Dx Respiratory SARS-CoV-2	31
QIAGEN, QIAsymphony DSP Virus/Pathogen Mini Kit	32
RBCBioscience, MagCore® Viral Nucleic Acid Extraction Kit	33
Roche, Cobas® Liat SARS-COV-2/FluA/FluB	34
Roche, Cobas® SARS-CoV-2: Qualitative assay for use on the cobas 6800/8800 Systems	35
Roche, DNA and Viral NA Large Volume kit	36
Roche, DNA and Viral NA Small Volume kit	37
Roche, MagNA Pure LC Total Nucleic Acid Isolation Kit	38
Seegene, STARMag 96x4 Universal Cartridge Kit	39
Siemens, VERSANT Sample Preparation 1.0 Reagents	40
TANBead, Nucleic Acid Extraction kit	41
Zeesan, lab-aid Virus RNA extraction kit	42

PCR/NAAT methods	
**Tests with 1 target gene**	
Abbott, ID NOW™ COVID-19	AA
BGI, BGI nCoV-19 detection kit	AB
Biotecon Diagnostics, microproof SARS-CoV-2 Identification Kit	AC
Bosch, Vivalytic SARS-CoV-2	AD
Gold Standard Diagnostics, NovaPrime SARS-CoV-2 (COVID-19)	AE
Hologic, Aptima® SARS-CoV-2 assay	AF
Hologic, Panther Fusion® SARS-CoV-2 assay	AG
N2-gene, Lu et al. 2020	AH
N-gene, Corman et al. 2020	AI
ORF1a/b (LAMP assay)	AJ
r-Biopharm, RIDA®GENE SARS-CoV-2 real-time PCR Kit	AK
RdRp-gene, Corman et al. 2020	AL
RdRP-gene, Corman et al. 2020 (adapted)	AM
Sarbeco E-gene, Corman et al. 2020	AN
Siemens, FTD Sars-CoV-2 assay	AO
TIB-MolBiol, LightMix® Modular Sarbecovirus E-gene, CE	AP
TIB-MolBiol, SARS-CoV-2	AQ
**Tests with 2 target genes**	
Abbott, ALINITY m SARS-COV-2 ASSAY	AR
Abott, M2000 SP	AS
Altona Diagnostics, Altona Realstar SARS-CoV-2 RT PCR	AT
BD, SARS-CoV-2 kit for BD MAX	AU
BioMerieux Diagnostics, Respiratory panel 2.1 plus	AV
Cepheid, Xpert® Xpress SARS-CoV-2	AW
Cepheid, Xpert® Xpress SARS-CoV-2/Flu/RSV	AX
Certest, VIASURE SARS-CoV-2 Real Time PCR	AY
Chemagic, SARS-CoV-2 RT-q PCR Perkin Elmer	AZ
Genmark, ePlex Respiratory Pathogen Panel 2	BA
Pathofinder, RealAccurate® Quadruplex SARS-CoV-2 PCR Kit	BB
PIIM, AmpliGnost E-Gen and RdRP-Gen qPCR	BC
Procomcure Biotech, PhoenixDx® SARS-CoV-2 Multiplex IVD	BD
QIAGEN, NeuMoDx™ Flu A-B/RSV/SARS-CoV-2 Vantage Test	BE
QIAGEN, QIAstat-Dx Respiratory SARS-CoV-2	BF
QIAGEN, SARS-CoV-2 N1+N2 Assay	BG
Roche, Cobas® Liat SARS-CoV-2/FluA/FluB	BH
Roche, Cobas® SARS-CoV-2: Qualitative assay for use on the Cobas 6800/8800 Systems	BI
Sarbeco E-gene, Corman et al. 2020; N1-gene, Lu et al. 2020	BJ
Sarbeco E-gene, Corman et al. 2020; N-gene, Lu et al. 2020	BK
Sarbeco E-gene, Corman et al. 2020; RdRP-gene, Corman et al. 2020	BL
Sarbeco E-gene, Corman et al. 2020; RdRP-gene, Corman et al. 2020 (adapted)	BM
Sarbeco E-gene, Corman et al. 2020; RdRP-gene, Lu et al. 2020	BN
Xiamen Zeesan Biotech Co., Ltd., SARS-CoV-2 Test Kit (Real-time PCR)	BO
**Tests with 3 target genes**	
Applied Biosystems, TaqPath COVID-19 Multiplex Diagnostic Solution (CE-IVD)	BP
ELITechGroup, GeneFinder COVID 19 PLUS Real Amp Kit	BQ
PCR Biosystems, qPCRBIO Probe 1-Step Virus Detect	BR
Sarbeco E-gene, Corman et al. 2020; N1-gene, Lu et al. 2020; N2-gene, Lu et al. 2020	BS
Seegene, Allplex 2019-nCov Assay	BT
**Tests with 4 target genes**	
Seegene, Allplex SARS-CoV-2 Assay	BU

Supplementary Figure 5. A flow diagram showing all workflows reported that have tested the EQA panel 2 with extraction method, PCR test, the number of target genes used, and the final score achieved by each workflow. In the alluvial plot PCR tests using one target gene are depicted in blue, PCR tests using two target genes are shown in red, PCR tests using three target genes are shown in green and PCR tests using four target genes are shown in purple. The color of trails per workflow are based on the score obtained for EQA panel 2. All workflows receiving scores below 6 are grouped in <6.

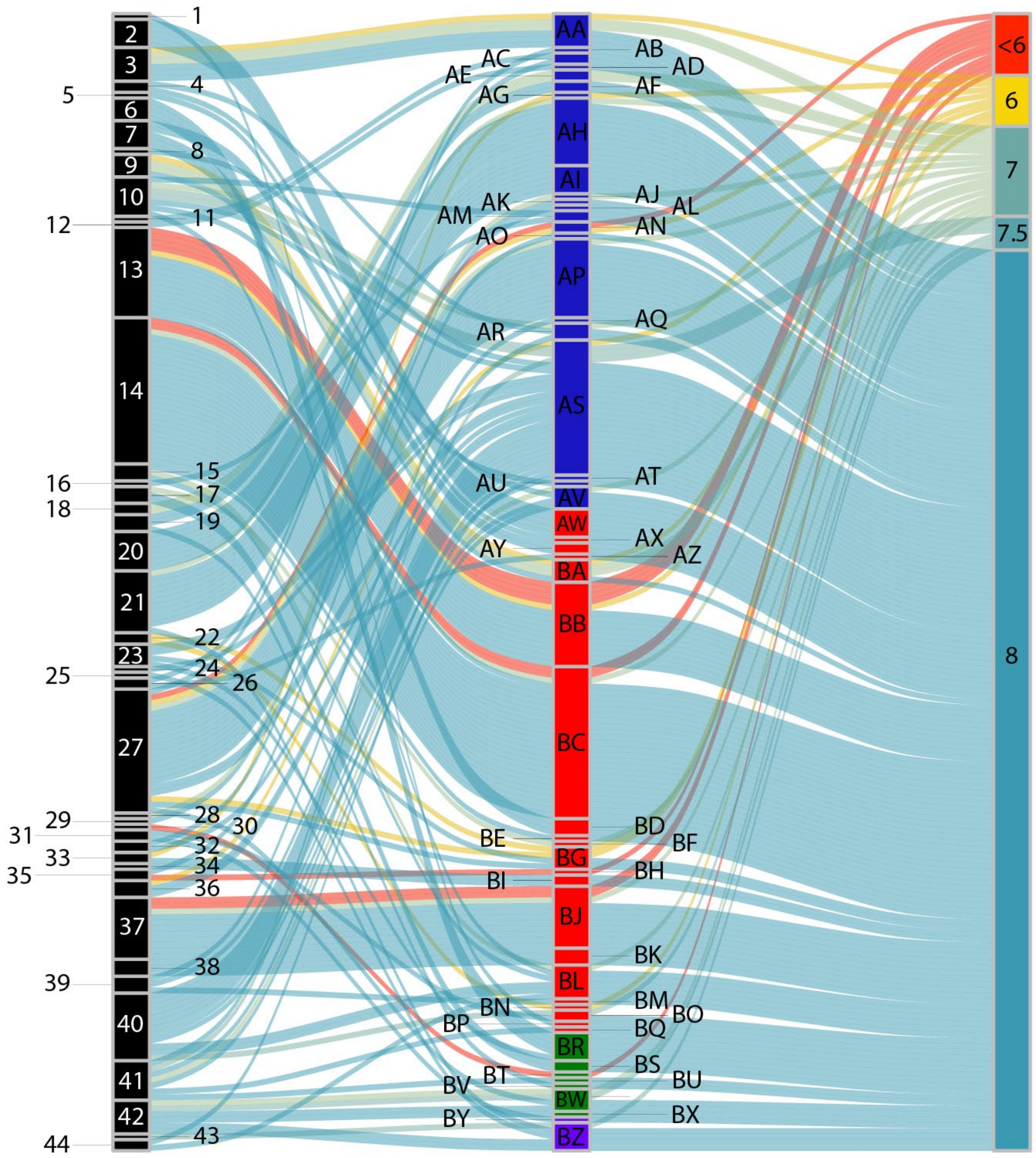
1
2
3
4
5
6
7
8
9
10
11
12
13
14
15
16
17
18
19
20
21
22
23
24
25
26
27
28
29
30
31
32
33
34
35
36
37
38
39
40
41
42
43
44
AA
AB
AC
AD
AE
AF
AG
AH
AI
AJ
AK
AL
AM
AN
AO
AP
AQ
AR
AS
AT
AU
AV
AW
AX
AY
AZ
BA
BB
BC
BD
BE
BF
BG
BH
BI
BJ
BK
BL
BM
BN
BO
BP
BQ
BR
BS
BT
BU
BV
BW
BX
BY
BZ
<6
6
7
7.5
8

RNA isolation methods	
A&A Biotechnology, MagnifiQ TM RNA buffer kit	1
Abbott, ALINITY m SARS-COV-2 ASSAY	2
Abbott, ID NOW™ COVID-19	3
Abclonal, ANDiS Viral Nucleic RNA Auto Extraction & Purification Kit	4
Altona Diagnostics, AltoStar® Purification Kit 1.5	5
Applied Biosystems, MagMAX TM Viral/Pathogen II Nucleic Acid Isolation Kit	6
BD, BD MAX ExK TNA-3	7
Bioecho, EchoLUTION viral RNA/DNA Swab 96 Kit	8
BioMerieux Diagnostics, Respiratory panel 2.1 plus	9
BioMerieux, NUCLISENS® easyMAG®	10
Biotecon Diagnostics, Magnetic Preparation Kit VI	11
Bosch, Vivalytic SARS-CoV-2 RT-PCR/NAAT	12
Cepheid, Xpert® Xpress SARS-CoV-2	13
Cepheid, Xpert® Xpress SARS-CoV-2/Flu/RSV	14
Chemagen, Chemagic Viral DNA/RNA 300 kit H96	15
Diagenode Diagnostics, MagDEA Dx cartridges	16
ElitechGROUP, ELITe InGenius SP 200	17
GenMark Diagnostics, ePlex® RP2	18
Goldstandard Diagnostics, NovaPrime RNA Extraction AE1	19
Hologic, Aptima® SARS-CoV-2 Assay	20
Hologic, Panther Fusion® SARS-CoV-2 assay	21
In-house	22
Life Technologies, MagMax pathogen RNA/DNA kit	23
Magcore, MagCore® Viral Nucleic Acid Extraction Kit	24
Magtivio, MagSi-NA Pathogens	25
MGI, MGIEasy Nucleic Acid Extraction Kit	26
Molgen, PurePrep Pathogens	27
MSE Group, SmartAmp SARS-CoV-2 RNA Detection Test Kit	28
NeuMoDx™, Flu A-B/RSV/SARS-CoV-2 Vantage Test Strip	29
Procomcure Biotech, SphaeraMag DNA/RNA Isolation Kit - prefilled96	30
Promega, Maxwell RSC Blood DNA kit	31
QIAGEN, NeuMoDx™ SARS-CoV-2 Assay	32
Qiagen, QIAamp Viral RNA Mini kit	33
Qiagen, QIAprep& Viral RNA UM Kit	34
Qiagen, QIAstat-Dx Respiratory SARS-CoV-2	35
Qiagen, QIAsymphony DSP Virus/Pathogen Midi Kit	36
Roche, Cobas® Liat SARS-COV-2/FluA/FluB	37
Roche, Cobas® SARS-CoV-2: Qualitative assay for use on the cobas 6800/8800 Systems	38
Roche, DNA and Viral NA Large Volume kit	39
Roche, DNA and Viral NA Small Volume kit	40
Roche, MagNA Pure LC Total Nucleic Acid Isolation Kit	41
Seegene, STARMag 96x4 Universal Cartridge Kit	42
Seegene, STARMag 96x4 Viral DNA/RNA 200 C Kit	43
Siemens, VERSANT Sample Preparation 1.0 Reagents	44

PCR/NAAT methods	
Abbott, ID NOW™ COVID-19	AA
BGI, Real-Time Fluorescent RT-PCR Kit for Detecting SARS-CoV-2	AB
Biotecon Diagnostics, microproof SARS-CoV-2 Identification Kit	AC
Bosch, Vivalytic SARS-CoV-2 RT-PCR/NAAT	AD
GenMark Diagnostics, ePlex® RP2	AE
Gold Standard Diagnostics, NovaPrime SARS-CoV-2 (COVID-19)	AF
Hibergene, HG COVID-19	AG
Hologic, Aptima® SARS-CoV-2 Assay	AH
Hologic, Aptima® SARS-CoV-2 Assay	AH
Hologic, Panther Fusion® SARS-CoV-2 assay	AI
MSE Group, SmartAmp SARS-CoV-2 RNA Detection Test Kit	AJ
NeuMoDx, Flu A-B/RSV/SARS-CoV-2 Vantage Test Strip	AK
N-gene, Jernigan 2020	AL
N-gene, Lu et al. 2020	AM
ORF1a/b (LAMP assay)	AN
Qiagen, QIAstat-Dx Respiratory SARS-CoV-2	AO
r-Biopharm, RIDAGENE SARS-CoV-2 RUO	AP
RdRp-gene (unknown)	AQ
Sarbeco E-gene (unknown)	AR
Sarbeco E-gene, Corman et al. 2020	AS
S-gene (unknown)	AT
Siemens, FTD Sars-CoV-2 assay	AU
TIB-MolBiol, LightMix® Modular Sarbecovirus E-gene, CE	AV
Abbott, ALINITY m SARS-COV-2 ASSAY	AW
Altona, Altostar SARS-CoV-2 RT PCR	AX
BD, SARS-CoV-2 kit for BD MAX	AY
BGI, Real-Time Fluorescent RT-PCR Kit for Detecting SARS-CoV-2	AZ
BioMerieux Diagnostics, Respiratory panel 2.1 plus	BA
Cepheid, Xpert® Xpress SARS-CoV-2	BB
Cepheid, Xpert® Xpress SARS-CoV-2/Flu/RSV	BC
Certest, VIASURE SARS-CoV-2 Real Time PCR	BD
Chemagic, SARS-CoV-2 RT-q PCR Perkin Elmer	BE
ELITechGroup, GeneFinder COVID 19 PLUS Real Amp Kit	BF
PathoFinder, RealAccurate® Quadruplex SARS-CoV-2 PCR Kit	BG
Qiagen, QIAstat-Dx Respiratory SARS-CoV-2	BH
Qiagen, SARS-CoV-2 N1+N2 Assay Kit	BI
Roche, Cobas® Liat SARS-CoV-2/FluA/FluB	BJ
Roche, Cobas® SARS-CoV-2: Qualitative assay for use on the cobas 6800/8800 Systems	BK
Sarbeco E-gene, Corman et al. 2020; N1-gene, Lu et al. 2020	BL
Sarbeco E-gene, Corman et al. 2020; N-gene, Corman et al. 2020	BM
Sarbeco E-gene, Corman et al. 2020; N-gene, Lu et al. 2020	BN
Sarbeco E-gene, Corman et al. 2020; RdRP-gene, Corman et al. 2020 (adapted)	BO
Siemens, FTD Sars-CoV-2 assay	BP
TIB-MolBiol, SARS-CoV-2	BQ
Applied Biosystems, TaqPath COVID-19 Multiplex Diagnostic Solution (CE-IVD)	BR
ELITechGroup, GeneFinder COVID 19 PLUS Real Amp Kit	BS
Procomcure Biotech, PhoenixDx® SARS-CoV-2 Multiplex IVD	BT
Sarbeco E-gene, Corman et al. 2020; N1-gene, Lu et al. 2020; N2-gene, Lu et al. 2020	BU
Sarbeco E-gene, Corman et al. 2020; N1-gene, Lu et al. 2020; S-gene (unknown)	BV
Seegene, Allplex™ 2019-nCoV Assay	BW
Seegene, Allplex™ SARS-CoV-2/FluA/FluB/RSV Assay	BX
del69/70 (unknown); E484K (unknown); N501Y (unknown); L452R (unknown)	BY
Seegene, Allplex™ SARS-CoV-2 Assay	BZ

Supplementary Figure 6. A flow diagram showing all workflows reported that have tested the EQA panel 3 with extraction method, PCR test, the number of target genes used, and the final score achieved by each workflow. In the alluvial plot PCR tests using one target gene are depicted in blue, PCR tests using two target genes are shown in red, PCR tests using three target genes are shown in green and PCR tests using four target genes are shown in purple. The color of trails per workflow are based on the score obtained for EQA panel 3. All workflows receiving scores below 6 are grouped in <6.
